# Gibberellins Producing Endophytic Fungus *Porostereum spadiceum* AGH786 Rescues Growth of Salt Affected Soybean

**DOI:** 10.3389/fmicb.2017.00686

**Published:** 2017-04-20

**Authors:** Muhammad Hamayun, Anwar Hussain, Sumera A. Khan, Ho-Youn Kim, Abdul L. Khan, Muhammad Waqas, Muhammad Irshad, Amjad Iqbal, Gauhar Rehman, Samin Jan, In-Jung Lee

**Affiliations:** ^1^Department of Botany, Abdul Wali Khan University MardanMardan, Pakistan; ^2^School of Applied Biosciences, Kyungpook National UniversityDaegu, South Korea; ^3^UoN Chair of Oman’s Medicinal Plants and Marine Natural Products, University of NizwaNizwa, Oman; ^4^Department of Agriculture, Abdul Wali Khan University MardanMardan, Pakistan; ^5^Department of Zoology, Abdul Wali Khan University MardanMardan, Pakistan; ^6^Department of Botany, Islamia College University PeshawarPeshawar, Pakistan

**Keywords:** endophytic fungi, *Porostereum spadiceum*, abscisic acid, gibberellins, isoflavonoids, soybean, salinity and biofertilization

## Abstract

In the pursuit of sustainable agriculture through environment and human health friendly practices, we evaluated the potential of a novel gibberellins (GAs) producing basidiomycetous endophytic fungus *Porostereum spadiceum* AGH786, for alleviating salt stress and promoting health benefits of soybean. Soybean seedlings exposed to different levels of NaCl stress (70 and 140 mM) under greenhouse conditions, were inoculated with the AGH786 strain. Levels of phytohormones including GAs, JA and ABA, and isoflavones were compared in control and the inoculated seedlings to understand the mechanism through which the stress is alleviated. Gibberellins producing endophytic fungi have been vital for promoting plant growth under normal and stress conditions. We report *P. spadiceum* AGH786 as the ever first GAs producing basidiomycetous fungus capable of producing six types of GAs. In comparison to the so for most efficient GAs producing *Gibberella fujikuroi*, AGH786 produced significantly higher amount of the bioactive GA_3_. Salt-stressed phenotype of soybean seedlings was characterized by low content of GAs and high amount of ABA and JA with reduced shoot length, biomass, leaf area, chlorophyll contents, and rate of photosynthesis. Mitigation of salt stress by AGH786 was always accompanied by high GAs, and low ABA and JA, suggesting that this endophytic fungus reduces the effect of salinity by modulating endogenous phytohormones of the seedlings. Additionally, this strain also enhanced the endogenous level of two isoflavones including daidzen and genistein in soybean seedlings under normal as well as salt stress conditions as compared to their respective controls. *P. spadiceum* AGH786 boosted the NaCl stress tolerance and growth in soybean, by modulating seedlings endogenous phytohormones and isoflavones suggesting a valuable contribution of this potent fungal biofertilizer in sustainable agriculture in salt affected soils.

## Introduction

Plant–microbe interactions are among the vital processes that are not only essential for the survival of both the partners but also important in functioning of agricultural system. Among the plant interacting microbes, endophytic fungi have gained marvelous attention of plant biologists due to their mutualistic interactions with the host crops. Much of the attention has been focused on phytostimulation under environmental stress by these organisms in order to devise the most suitable biofertilizers (Baltruschat et al., 2008; Miransari, 2011). Plants hosting such beneficial fungi undergo significant changes in their key physiological aspects, including phytohormones balance, root exudation, composition of mineral nutrients, and physical modification in soil (Richardson, 2009; Kusari et al., 2013). These changes enable host plant to withstand different abiotic and biotic stresses which ultimately enhance their overall fitness (Chandanie et al., 2006; Khan et al., 2009).

An important stress among abiotic stresses is salinity, as it is responsible for diverting plant metabolism to deposit organic solutes so as to maintain turgor pressure. Such osmolytes including proline have been shown to function as osmoprotectants (Munns and Tester, 2008). Plants exposed to salinity have to cope with osmotic and ionic stresses to which they respond by induction of a defense response. Salt stressed plants are characterized by aggregation of compatible osmolytes, which induce additional water uptake in plant root from surrounding, to buffer the immediate effect within plant cell (Bartels, 2005; Misra and Gupta, 2005; Verbruggen and Hermans, 2008). In leguminous plants including soybean, salinity is correlated with poor yield and reduction in plant growth (Dinler et al., 2014). The severity of the situation is enhanced when salt stress interferes with the symbiotic association of rhizobia and roots of legumes (Dardanelli et al., 2010; Mhadhbi et al., 2014). Fungal endophytes may be of special significance because of their ability to colonize legumes and help their partner to survive under extreme environmental conditions by secreting beneficial secondary metabolites.

Soybean [Glycine max (L.) Merril] is an important legume cultivated worldwide for its oil and protein rich grains. The crop has been known as a rich source of beneficial endophytic fungi improving its fitness to withstand environmental stresses including salinity (Pimentel et al., 2006; Khan et al., 2011). About 187 species of fungal endophytes have been reported from different parts of soybean (Fernandes et al., 2015) which belong to different taxonomic groups including Ascomycota and Basidiomycota (de Souza Leite et al., 2013). Soybean leaves were comparatively richer in fungal endophytes than its roots.

Isoflavones are important class of compounds having twofold importance, acting as signals thereby mediating root interaction with fungi. Additionally, isoflavones may improve survival of soybean by acting as antimicrobial agents (Dakora and Phillips, 1996; Messina, 2010). Isoflavones have useful implications in human health as they decrease risks of cancers (breast, colon, and prostate), premature menopause and cardiovascular disorders (Anderson and Keifer, 1997; Allred et al., 2004; Phommalth et al., 2008). Information on the modulation of soybean endogenous isoflavone and proline content by phytostimulant fungi has been scarce. We preferred isoflavones determination in salt affected soybean because they act as important regulators of root nodulation, symbiotic interactions and plant defense thereby favoring plant to withstand environmental pressure. Silencing of soybean biosynthesis gene has been shown to increase susceptibility to *Phytophthora sojae* (Subramanian et al., 2005). Additionally, isoflavones are greatly valued in human health. Because of these properties, selection or development of soybean cultivars with higher isoflavone content has been focused in the past (Phommalth et al., 2008). Similarly, *in planta* GAs enhancement by fungal endophytes may promote the activity of phenyl alanine ammonia lyase, one of the key enzyme in isoflavone biosynthesis pathway, thereby enhancing synthesis of isoflavones by plant itself (Barnes and Jones, 1984).

Likewise, in stressed plants, endogenous level of phytohormones has been widely investigated, even then the role of endophytic fungi in alleviation of biotic and abiotic stresses by modulating phytohormones has been least explored. Similarly, the ability of endophytic fungi to produce GAs in pure culture and promote plant growth has widely been reported (Hamayun et al., 2009; Khan et al., 2009, 2011). However, use of endophytic fungi to recover plants from the damaging effects of salinity is limited to a few works (Waller et al., 2005; Khan et al., 2012). Most of the plant growth promoting endophytic fungi belong to the group of sac fungi known as Ascomycota (Khan et al., 2009; Zhou et al., 2015). However, members of club fungi (Basidiomycota) has also been shown to exist as endophytes in plant tissues and promote growth by different mechanisms (Waller et al., 2005). Among several mechanisms of phytostimulation is direct growth promotion by the secretion of phytohormones including gibberellins (Hamayun et al., 2009; Khan et al., 2009). However, GAs and other phytohormones may also modulate plant physiology making them resistant to different environmental stresses (Waqas et al., 2012, 2015). Several members of Ascomycota has been known to live as plant endophytes and promote plant growth by producing GAs (Ansari et al., 2015; Leitão and Enguita, 2016). However, to date none of the basidiomycetous fungal endophyte has been reported to produce GAs. Current study was proposed to evaluate the phytostimulatory potential of a novel GAs producing endophyte member of Basidiomycota, *Porostereum spadiceum* AGH786, and its ability to modulate endogenous contents of GAs, ABA, JA, and isoflavones.

## Materials and Methods

### Isolation of Endophytic Fungi from Soybean Roots

For isolation of endophytes, 27 days old soybean (cv. Hwangkeumkong), grown in 5.5 L plastic pots under greenhouse conditions were collected. The plants were grown in horticultural soil composed of peat moss (13–18%), perlite (7–11%), coco-peat (63–68%) and zeolite (6–8%), while the macro-nutrients were present as follows: NH_4_+∼90 mg/L; NO_3_-∼205 mg/L; P_2_O_5_∼350 mg/L, and K_2_O∼100 mg/L. The pH was neutral (pH 7).

To isolate endophytic fungi, roots of the selected plants were washed with running tap water and treated with Tween 80 solution. Thoroughly, washed roots of 10 soybean plants were cut to obtained 10 segments (0.5 cm) per plant making a total of 100 root segments which were then surface sterilized in perchloric acid (Khan et al., 2008). After surface sterilization, the sections were imprinted on Hagam medium plates to check their sterility (Berg et al., 2005). After incubation of 7 days, the sterile root segments were subjected to endophytes isolation procedure. The sterilized root sections were then injured by pressing against glass slide to expose internal tissues and inoculated on agar plates containing Hagem medium (5 pieces/plate) supplemented with streptomycin (for avoiding bacterial contamination). The plates were then incubated at 25°C till fungal colonies emerged from the plant sections (Khan et al., 2009). After the emergence of fungal spots, subculturing was done by shifting distinctly different fungal spots on to Hagem and PDA medium plates and allowing them to grow for 7 days at the above mentioned temperature. Subculturing was repeated until the establishment of pure cultures which were stored on PDA slants under mineral oil at -20°C.

### Screening the Endophytic Fungi for Beneficial Traits of Plant Growth Promotion

Low GAs dwarf cultivar of rice (Waito-c rice) was used to screen the isolated *fungal strains* for GAs production and Phytostimulation. Rural Development Administration (RDA) Korea kindly provided seeds of Waito-c. Fungal cultures were grown in broth culture containing Czapek medium for 7 days at 30°C and 120 rpm. Fungal biomass was separated from culture medium by centrifugation (10000 rpm) at 4°C for 15 min. Culture supernatant and fungal biomass were separately lyophilized. Pellet containing fungal biomass was used to identify fungal isolates and the lyophilized supernatant was resuspended in sterilized distilled water making its volume to 1 mL. Surface sterilized healthy seeds of Gas deficient rice were dipped in 20 ppm uniconazol solution for 24 h. The seedlings were grown in 0.8% water-agar medium under axenic conditions in growth chamber (photoperiod of 14 h light and 10 h dark; light intensity 1000 μmm^-2^s^-2^ Natrium lamps and relative humidity 60–70%). Fungal culture supernatant (10 μL) was applied on the apices of seedlings at 2-leaf stage. Seedlings treated with distilled water and supernatant of *Gibberella fujikuroi* culture were used as negative and positive controls respectively. Seedlings were harvested 1 week post-supernatant treatment and different growth parameters were noted.

### Fungal Identification and Phylogenetic Analysis

Molecular approach was adopted to identify the selected fungal strain AGH786. Protocol of Khan et al. (2008) was followed to isolate fungal genomic DNA and carry out PCR for the amplification of internally transcribed region of 18S rRNA gene using primers ITS-1 and ITS-4 (Khan et al., 2008). The ITS regions was sequenced and the obtained sequence was subjected to BLASTn^1^ for sequence homology estimation. The sequences obtained as a result of homology search, transformed to maximum parsimony tree through MEGA 4 (Tamura et al., 2007).

### Screening Isolates for Halotolerance

*Porostereum spadiceum* AGH786 was grown in Czapek broth supplemented with different concentrations (50–500 mM) of salt (NaCl). After incubation of 7 days in shaking flasks, fungal mycelia were filtered out and subjected to fresh and dry weight determination. Czapek medium composed of standard recipe (Krzyśko-Lupicka et al., 1997) was used as control.

### Soybean–endophytes Interaction

Surface sterilized seeds of soybean (*Glycine max* L. Hwangkeum) were grown in sterilized pots (15 L) containing autoclaved (once) horticultural soil (composition described above) and maintained axenically (Misra and Dwivedi, 2004). Pots were kept for 3 weeks under conditions mentioned above in randomized complete block design. The seedlings received two treatments with each treatment having two levels constituting four different sets of seedlings, e.g., seedlings receiving neither salt nor endophytic association (NEA), seedlings receiving both endophytic association and salt stresses (EAS), and seedlings with either endophytic association (EA) and or salt stress (NEAS) only.

To prepare inoculum of fungal strains, mycelium was harvested by centrifugation at 5000 × *g* and 4°C for 15 min from 7 days old fungal culture grown in Czapek broth (250 mL) at 30°C. To each pot, soil mixed with 50 mg of crushed fungal mycelium was added and the pots were kept in controlled environment for 3 days under conditions mentioned elsewhere to acclimatize fungal isolate.

Twenty one days old soybean seedlings were exposed to moderate (70 mM) and high (140 mM) NaCl stress. Seedlings were watered with 1400 mL of salt solution or distilled water (control) for 7 days.

### Analysis of Plant Growth Attributes

Soybean seedlings were harvested to investigate shoot length and biomass. Chlorophyll was estimated through chlorophyll meter (SPAD-502 Minolta, Japan) and rate of photosynthesis of the selected leaves was also measured (ADC Bioscientific LCi analyzer; Model31655 UK). Area of mature leaves were measured with Laser Leaf Area meter (CI-203 model, CID, Inc., USA).

### Determination of Phytohormones

Phytohormones including GAs were determined in fungal culture filtrate collected after growing AGH786 for 7 days in Czapek broth culture under conditions mentioned above. To know the endogenous status of phytohormones in seedlings, endogenous GAs, ABA, and JA were determined in the seedlings exposed to different treatments.

### Determination of GAs

To determine fungal GAs, 30 mL of culture filtrate was used to extract GAs according to Khan et al. (2009). For determining endogenous GAs of soybean, seedlings were crushed under liquid nitrogen to fine powder. Gibberellins were extracted and quantified by using 0.5 g fine powder. Instrument used for the determination of GA was gas chromatograph (Hewlett-Packard 6890, 5973N mass selective detector) with HA-1 capillary column (30 m × 0.25 mm i.d. 0.25 μm film thickness) and oven with a range of temperature, i.e., initial 60°C for 1 min, elevated to 200°C at the rate of 15°C min^-1^, then to 285°C at the rate of 5°C min^-1^). The carrier gas (helium) was maintained under a pressure of 30 kPa. Gas chromatography and mass selective detector were directly connected with 280°C temperature of furnace, with ionizing voltage and dwell time of 70 eV and 100 ms respectively. Full scan mode (the first trial) and three major ions of supplemented [^2^H_2_], GAs internal standards (the second trial) and the endogenous gibberellins were monitored simultaneously. The amount of endogenous GA_4_, GA_9_, and GA_12_ (ng/g of dry weight) were calculated from the peak area ratios of 284/286, 298/300, and 300/360 respectively. The analysis was repeated two times.

### Determination of ABA

The level of endogenous free ABA was determined in soybean shoots as described (Yoon et al., 2009). Soybean shoots (0.5 g) were ground to powder and then extracted with mixture (30 mL) composed of isopropanol and glacial acetic acid in 19:1 and 100 ng of [^2^H_6_]-ABA as internal standard. After filtration, the solvent was evaporated from the filtrate at reduced pressure using rotary evaporator. Further purification was exactly similar as described previously (Yoon et al., 2009). The pure sample was derivitized by treatment with diazomethane and analyzed by GC–MS SIM (6890N network GC system, equipped with 5973 network mass selective detector; Agilent Technologies, Palo Alto, CA, USA). The Lab-Base (ThermoQuset, Manchester, UK) data system software was employed to monitor responses to ions of m/e 162 and 190 for Me-ABA and 166 and 194 for Me-[^2^H_6_]-ABA.

### Determination of JA

To determine the contents of JA in soybean shoots, 0.1 g of fine powder was extracted with a mixture of 50 mM citric acid and acetone (30:70, v/v) containing 20 ng internal standard, [*9*, *10*-^2^H2]*Dihydro*-*JA*. To get rid of the volatile fatty acids, the extracts were left overnight for evaporation at room temperature. The aqueous phase obtained was passed through filter and extracted thrice with 10 mL aliquots of diethyl ether. The three aliquots of diethyl ether were pooled and subjected to a solid phase extraction column composed of 500 mg of sorbent, aminopropyl. The column was then washed with a 7 mL mixture containing 2-propanol and trichloromethane in 2:1, v/v. The column was eluted with 10 mL mixture of acetic acid and diethyl ether in 98:2, v/v. From the eluate containing JA, the solvent was evaporated and to the residue excess of diazomethane was added to derivatize JA for analysis by GC/MS (6890N network GC system, and 5973 network mass selective detector; Agilent Technologies, Palo Alto, CA, USA). Sensitivity of the protocol was maximized by recording the spectra in the selected ion mode. Peak area produced by JA in the sample was compared with the standard to estimate its amounts (ng/g dry weight). Three different samples were used to repeat the analysis.

### Isoflavone Content Analysis

Previously established protocols were followed for the isolation and determination of isoflavones in soybean seedlings (Rostagno et al., 2003; Phommalth et al., 2008). Briefly, 10 mL of 80% EtOH was mixed with 0.2 g of dry powder and kept at 50°C for 1 h in ultrasonic bath (Kodo, Co., Korea). Afterward, the samples were shaken at 50°C and 150 rpm for 15 h. Syringe filteration through 0.45 μm filters was done to make the sample debris free and the filtrate (10 μL) was then eluted from HPLC (PerkinElmer series 200, USA) column (C18 column 4.6 mm × 150 mm; 5 μm) using gradient solutions viz. 0.1% of acetic acid and acetonitrile in deionized water keeping flow rate at 1.0 mL min^-1^ The UV detector was set to 260 nm for the detection of isoflavones in the eluant. Peak areas of the standards isoflavones genistein and daidzein (Sigma Chemical, Co, USA) were compared with that of the samples to estimate the quantity of the isoflavones.

### Statistical Analysis

Experiments were repeated three times with exception of GA determination which was performed twice. Means were compared by performing one way analysis of variance (ANOVA) and Duncan multiple range test (DMRT) at *p* = 0.05 using SPSS for windows.

## Results

### Screening Bioassay for Isolated Strains

**FIGURE 1:**
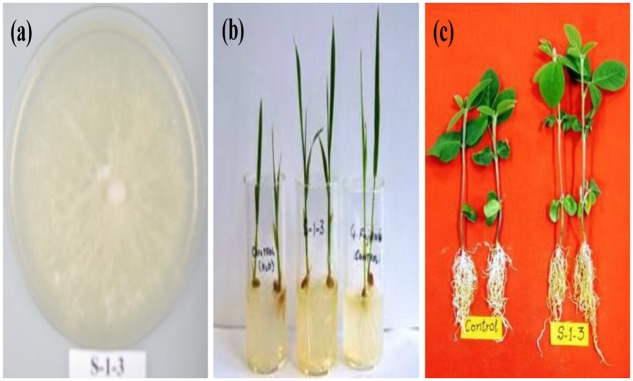
**Seven days old colony of the endophytic fungus *Porostereum spadiceum* AGH786 (labeled as S-1-3) isolated from soybean plants on Czapek agar (a)**, which restored growth of wito-c rice (**b** middle flask) like *Gibberella fujikuroi* (**b** right flask) and enhanced growth of soybean seedlings **(c)** grown under axenic conditions for 3 weeks.

Isolated endophytes were screened for GAs production by applying their culture supernatant on Waito-c rice seedlings and their performance was compared with that of wild strain of *G. fujikuroi*. For this purpose, 10 μL of resuspended lyophilized supernatant from individual strains were applied on shoot apex of 10 days old seedlings. Growth attributes of Waito-c rice seedlings were analyzed 1 week after supernatants application. A total of 42 strains were initially isolated and tested for plant growth promotion. Assay on Waito-c rice showed that 34 strains were able to promote the growth attributes of soybean and eight strains were inhibitors (data not shown). Based upon its excellent performance in terms of growth promotion of Waito-c rice, S-1-3 was selected for further study (**Figure 1**). During further study, name of the strain was changed to AGH786.

### Identification of AGH786

**FIGURE 2:**
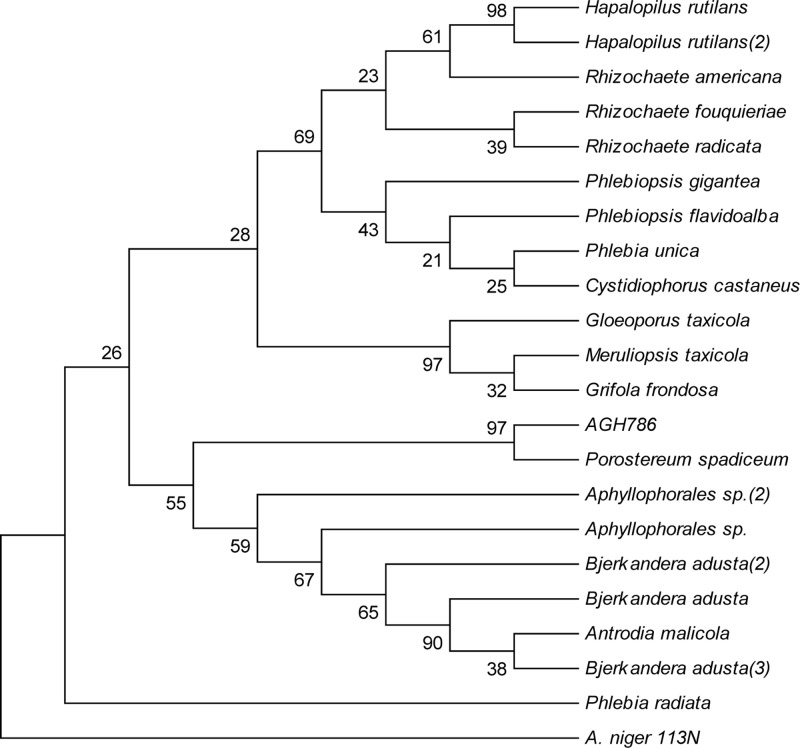
**Phylogenetic tree constructed by Neighbor-Joining method showing the evolutionary history of AGH786**. Ascomycetous *Aspergillus niger* 113N was used as outgroup.

Based upon BLAST results, sequence of 28S rDNA of AGH786 showed maximum homology (99%) with *P. spadiceum*. To confirm identity of the strain, its sequence was subjected to phylogenetic analysis (Khan et al., 2008). Phylogenetic consensus tree was constructed from 22 (21 reference and 1 clone) by using neighbor joining (NJ) method in MEGA 6 package (**Figure 2**). Our isolate AGH786 formed a clad with *P. spadiceum* supported by 99% boostrap value in the consensus tree. Combine results of sequence homology and phylogenetic analysis suggested that the strain AGH786 was *P. spadiceum*. Sequence of its 28S rDNA was submitted to GenBank under accession No. FJ808680.

### Characterization of Endophyte AGH786

#### Halotolerance of AGH786

**FIGURE 3:**
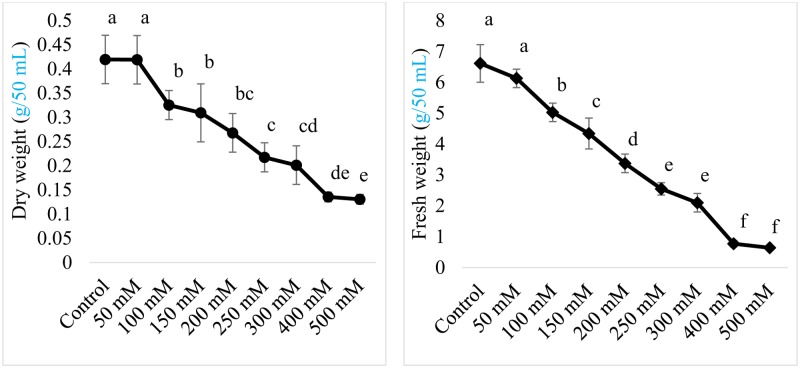
**Effect of salt (NaCl) concentration on the fresh and dry weight of endophytic strain AGH786 of *P. spadiceum***. The strain was grown for 7 days in shaking flasks containing 50 mL Czapek culture broth provided different concentrations of NaCl. Bars show standard error of mean and data points labeled with different letters are significantly different (*p* < 0.05).

Halotolerance of the isolated endophyte AGH786 was tested by exposing the strain to different concentrations of NaCl. Current results showed that both fresh and dry weight of AGH786 was not affected by elevated NaCl stress (50 mM) showing its ability to tolerate salinity stress (**Figure 3**). However, further increase in salt concentration imposed significant reduction in growth of AGH786 when its fresh and dry biomass in salt supplemented and control media were compared (**Figure 3**). A strong negative correlation was recorded between salt and fresh weight (*r* = -0.979; *p* < 0.01) and also between salt concentration and dry weight (*r* = -0.969; *p* < 0.01). Increasing concentration of salt to 150 mM reduced fresh and dry fungal biomass by 29 and 26% respectively as compared to the fresh and dry weight of fungi grown in control media. Minimal inhibitory concentration of NaCl was around 250 mM, reducing fresh and dry weight of this fungus by 50%. Further increase was detrimental to fungus growth, reducing fresh biomass from 6.6 mg (in control media) to 0.64 mg (in media containing 500 mM NaCl). Decrease in dry biomass was less sharp as 500 mM NaCl reduced it by only threefold as compared to control (**Figure 3**).

#### Free Amino Acids in Culture Filtrate of AGH786

Free amino acids in the rhizosphere are important source of nitrogen available to plants and they compete with microbes for obtaining free amino acids (Owen and Jones, 2001; Jones et al., 2005). Being important in plant growth, we observed whether this isolate can release free amino acids in the culture filtrate. Twenty seven different free amino acids were detected in the culture filtrate of this fungus (**Table 1**). Among different amino acids citrullin and *sarcosine were the most abundant types. Hydroxylysine, tyrosin, cystein*, glutamic acid, and aspartic acid were also released in handsome amount to the culture media.

**Table 1:** Free amino acids determined in the culture filtrate of *Porostereum spadiceum* AGH786 grown in Czapek media for 7 days.

S. no	Amino acid	Amount (μg mL^-1^)	S. no	Amino acid	Amount (μg mL^-1^)
1	Phosphoserine	119.14	15	Leucine	253.48
2	Urease	7.8	16	Tyrosine	466.07
3	Aspartic acid	473.42	17	Phenylalanine	12.06
4	Threonine	283.46	18	β-Amino isobutyric acid	20.91
5	Serine	311.85	19	γ-Amino-*n*-butyric acid	21.06
6	Glutamic acid	402.63	20	Ethanol amine	3.94
7	Sarcosine	628.34	21	Ammonia	1.63
8	α-Aminoadipic acid	21.74	22	Hydroxylysine	588.22
9	Glycine	12.67	23	1-Methylhistidine	38.36
10	Alanine	263.48	24	Histidine	160.42
11	Citrulline	664.83	25	Anserine	131.88
12	α-Amino-*n*-butyric acid	16.89	26	Arginine	8.44
13	Cystine	545.52	27	Proline	183.14
14	Isoleucine	8.51			

#### Determination of GAs in the Culture Filtrate of AGH786

**FIGURE 4:**
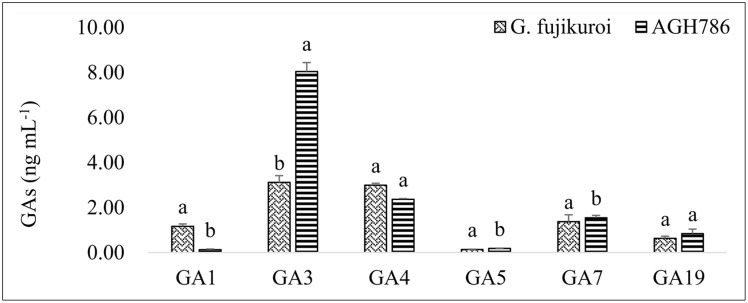
**Production of Gibbrellins (GAs) by *P. spadiceum* AGH786 and wild strain of *G. fujikuroi***. After 7 days of growth in Czapek broth, GAs were extracted from fungus culture filtrate and quantified through GC–MS/MS. Bars show standard error of mean and data points labeled with different letters are significantly different (*p* < 0.05).

Production of bioactive GAs in the culture filtrate of fungal isolate AGH786 was determined qualitatively and quantitatively. We could detect six different types of bioactive GAs, including *GA_1_*, *GA_3_*, *GA_4_*, *GA_5_*, *GA_7_*, and *GA_19_* (**Figure 4**). *GA_3_* was the most abundant type of the GAs and its production by AGH786 was more than twofold greater than its amount produced by wild type *G. fujikori*. So far, this is the first fungal endophyte producing *GA_3_* in greater amount than *G. fujikori*. However, the quantity of other GAs was either similar in both strains or greater in the filtrate of *G. fujikuri* than its amount in the culture filtrate of AGH786 (**Figure 4**). Bioactive *GA_3_* and *GA_4_* have been known to play exponential role in promoting plant growth (Eriksson et al., 2006) and higher growth of Waito-c rice seedlings by AGH786 supports our findings.

#### Soybean–endophyte Interaction

**FIGURE 5:**
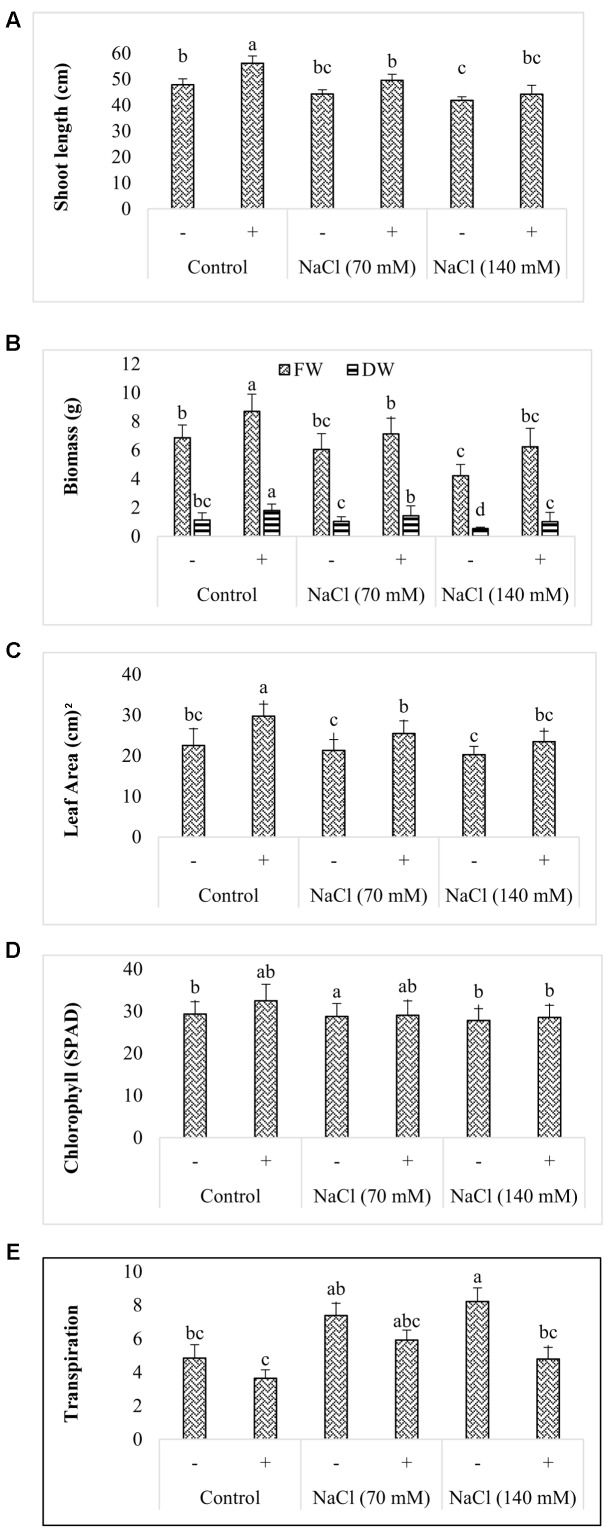
**Effect of *P. spadiceum* AGH786 on growth of soybean seedlings grown for 3 weeks under axenic conditions**. Growth parameters include **(A)** shoot length, **(B)** biomass, **(C)** leaf area, **(D)** chlorophyll, and **(E)** transpiration. Data are means of three replicates along with standard error bars. Mean bars labeled with different letters are significantly different (*p* < 0.05).

When co-cultured with AGH786, significant improvement was recorded in the growth of soybean seedlings. Soybean seedlings grown on soil with mixed AGH786 mycelium, showed higher shoot growth as compared to the plants grown without fungus (**Figure 5A**). However, shoot dry weight was not affected by the fungus indicating that AGH786 improved water absorption or its retention by the seedlings.

The isolate significantly enhanced fresh and dry biomass of seedlings in the absence of salt stress (**Figure 5B**). In comparison to control, fresh and dry biomass was increased by 26.8 and 57.8% respectively. Salt stress (70 mM NaCl) reduced fresh and dry biomass by 11.8 and 8.7% respectively when compared with that of control. Further decrease of 38.6 and 52.6% respectively in these parameters was observed by elevating salt concentration to 140 mM. Inoculation of salt stressed seedlings with AGH786 alliviated the stress shown by restoration of fresh and dry biomass to the level of control in such plants (**Figure 5B**). In comparison to control, the strain caused an improvement of more than 31% in leaf area of seedlings grown under normal conditions (**Figure 5C**). The same behavior of the strain was observed in salt stress seedlings. However, exposure to the stress did not affect the parameter significantly (**Figure 5C**). Increase in the chlorophyll content in the inoculated seedlings compared to the non-inoculated plants was statistically non-significant (**Figure 5D**). By contrast, transpiration rate was significantly reduced in seedlings cocultivated with the strains under normal as well as saline conditions (**Figure 5E**).

### Modulation of Endogenous Phytohormones in Soybean under Stress

#### GAs Analysis

**FIGURE 6:**
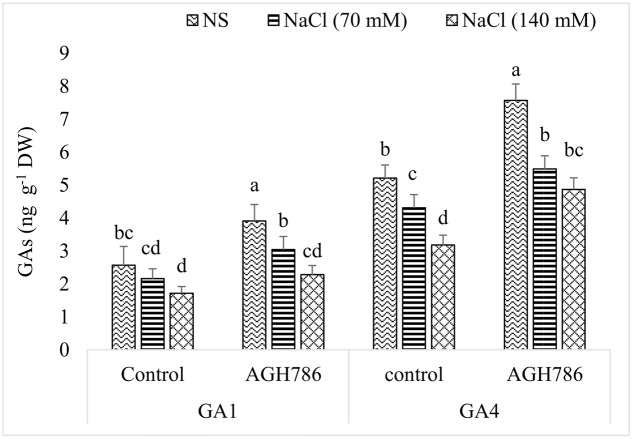
**Effect of endophytic fungus *P. spadiceum* AGH786 and different salt concentrations (70 and 140 mM) on the endogenous level of GAs in soybean seedlings axenically grown for 3 weeks**. Concentrations of GAs were determined by GC–MS/MS. Data are means of three replicates along with standard error bars. Mean bars labeled with different letters are significantly different (*p* < 0.05).

Bioactive GAs, i.e., GA_1_ and GA_4_ were determined because of their importance during salt stress. It was obvious that salt stress negatively regulated the synthesis of GA_1_ and GA_4_ in a dose dependent manner in seedlings grown with or without endophyte. However, in seedlings colonized by AGH786, production of GAs was always greater than that in their respective controls (**Figure 6**). For example, production of GA_1_ was 52% higher in control seedlings colonized by AGH786 than that of uncolonized seedlings. Similarly, in salt affected seedlings, 41 and 33% greater contents of endogenous GA_1_ was recorded in AGH786 treated seedlings exposed to 70 and 140 mM NaCl respectively when compared with their respective controls. In a similar fashion, endogenous level of GA_4_ was increased by 45% in seedlings grown in association with AGH786. In salt affected seedlings, synthesis of GA_4_ was enhanced by 27 and 53% in AGH786 treated seedlings exposed to 70 and 140 mM NaCl respectively when compared with their respective controls.

#### ABA Analysis

**FIGURE 7:**
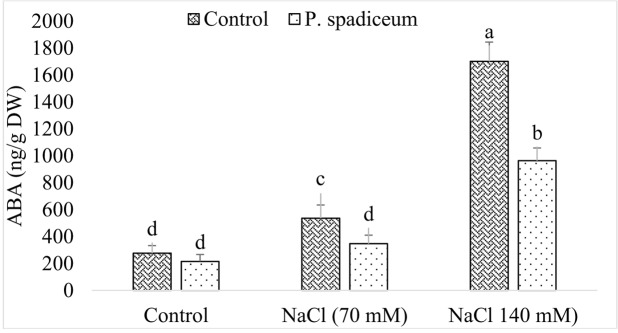
**Effect of endophytic fungus *P. spadiceum* AGH786 and different salt concentrations (70 and 140 mM) on the endogenous level of ABA in soybean seedlings**. Data are means of three replicates along with standard deviation bars. Mean labeled with different letters are significantly different (*p* < 0.05).

In the absence of NaCl, ABA contents in seedlings treated with fungal endophyte and untreated seedlings were almost similar. However, a sharp and dose dependent increase in the production of ABA was observed in seedlings exposed to salt stress (**Figure 7**). In comparison to control (not treated with AGH786), increase in ABA production was significantly less steep in seedlings colonized by AGH786 showing alleviation of the stress. Nevertheless, doubling the dose of salt from 70 nM to 140 mM enhanced ABA production in both endophyte associated and non-associated seedlings.

#### JA Analysis

**FIGURE 8:**
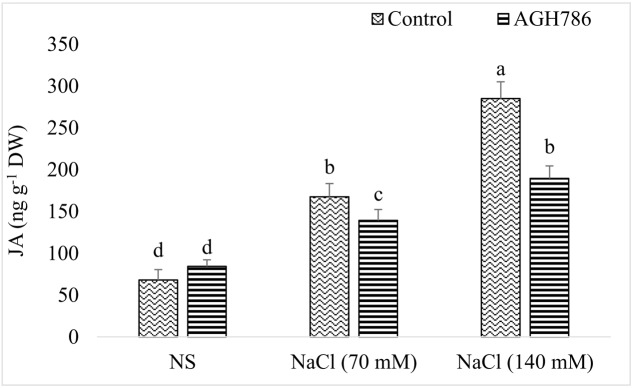
**Effect of endophytic fungus *P. spadiceum* AGH786 and different salt concentrations (70 and 140 mM) on the endogenous level of JA in soybean seedlings**. Data are means of three replicates along with standard error bars. Mean bars labeled with different letters are significantly different (*p* < 0.05).

In the absence of salt stress, JA content of soybean seedlings colonized by AGH786 was comparable to that of seedlings not associated with the endophyte (**Figure 8**). However, exposure to salt stress increased JA production by the seedlings in a dose dependent manner. More than twofold increase was recorded in the endogenous JA levels of seedlings treated with 70 mM NaCl as compared to that of the control which was reduced by 41% in seedlings colonized by AGH786. Similarly, doubling salt concentration in the media enhanced JA production by more than threefold when compared with control (seedlings grown in the absence of salt stress). However, colonization of AGH786 on the roots of the seedlings reduced JA contents down to half of the seedlings treated with this double dose of NaCl alone. Though endophyte treatment reduced JA production in salt affected seedlings, its amount was still higher than that of the control seedlings (seedlings grown in the absence of salt and endophyte).

#### Isoflavone Content Analysis

**FIGURE 9:**
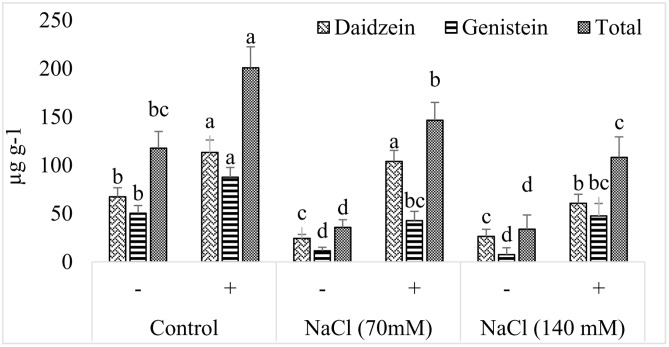
**Effect of halotolerant fungal endophyte (*P. spadiceum* AGH786) on the endogenous isoflavone (daidzein and genistein) contents of soybean grown under elevated salt stress**. Error bars represent ± standard error (of three replications). Mean bars labeled with different letters are significantly different at *p* < 0.05 analyzed by Duncan multiple range test.

Soybean plants are famous for the production of isoflavones, daidzein, and genistein which are used as markers of food quality. It was noted that production of isoflavones was greatly reduced in soybean upon exposure to salt stress (**Figure 8**). In case of daidzein, more than twofold reduction was recorded in salt seedlings exposed to the selected concentration of NaCl. However, increaing concentration of NaCl was not linked with any further decrease in the production of daidzein. Contrary to this, effect of salt on the production of genistein was dose dependent and more drastic (**Figure 9**). In the presence of 70 mM NaCl, genistein production was reduced by more than fourfold and further decrease was noted by doubling the amount of NaCl. In the absence of NaCl, AGH786 associated seedlings produced isoflavones in significantly greater amount as compared to that of control seedlings. Interestingly, AGH786 restored the production of isoflavones in salt affected seedlings too (**Figure 9**).

## Discussion

Soybean is an important legume cultivated worldwide for its oil and protein rich grains. However, its production is under threat due to ever increasing environmental stresses including salinity (Pimentel et al., 2006). Among the various strategies, use of endophytic fungi as biofertilizers are emerging as the most efficient tools for sustainable agricultural practices on salt affected areas (Radhakrishnan et al., 2014). Soybean has been known as a rich source of beneficial endophytic fungi improving its fitness to survive under stress (Khan et al., 2011). Soybean endophytes belong to diverse taxonomic groups such as Ascomycota and Basidiomycota (de Souza Leite et al., 2013). For soybean cultivation on salt affected soils, we attempted to isolate halotolerant endophytic fungus from Soybean itself having the potential to readily colonize its host and alleviate salt stress. One of the endophyte *P. spadiceum* strain AGH786, residing soybean root proved to be plant growth promoting, GAs producing (assayed on Waito-c rice) and halotolerant to NaCl. The GAs mutant Waito-c rice is an excellent system used to screen culture filtrate of endophytes for GAs and plant growth promotion (Potshangbam et al., 2017). Production of GAs is an important trait enabling endophytes to promote plant growth and mitigate salt stress (Hamayun et al., 2015). In addition to GAs, Several amino acids were also detected in the culture of this strain. Similarly, this strain not only enhanced soybean growth under control environment but also under salt stress.

Several endophytic fungi including *Aspergillus niger*, *A. flavus*, *Fusarium oxysporum*, *Penicillium funiculosum*, *P. corylophilum*, *Rhizopus stolonifer*, and *Paecilomyces formosus* have been reported to produce phytohormones including GAs and IAA (Khan et al., 2015; Deng and Cao, 2017). However, all the fungal species capable of GA production belong to ascomycetes, a group of ascus forming fungi. *P. spadiceum* strain AGH786 has become the first GAs producing fungal endophyte that belongs to the group Basidiomycota. Plants treated with endophytes are often healthier than those lacking such interaction (Khan et al., 2008; Larriba et al., 2015), which in most cases is attributed to the endophyte secretion of phytohormones such as GAs (Waqas et al., 2012). In endophyte–host symbioses, secondary metabolites may be a contribution of the endophytic partner for such mutualistic relationship (Shi et al., 2009; Kusari et al., 2013).

Phytohormones signaling and crosstalk is of great significance in plant growth and development under normal and stress conditions. Under salinity stress, plant protects itself by producing extra amount of ABA which mediate stomatal closure to minimize water loss and then mediates stress damage (Chaves et al., 2009). According to one theory, CKs: ABA ratios in xylem sap controls stress signaling (Tran et al., 2010; Nishiyama et al., 2011). During salt stress, ABA production shoots up which is capable of repressing the expression of isopentenyl transferase gene encoding a rate limiting enzyme in the biosynthesis of CKs, suppressing amount and signaling of CKs (Nishiyama et al., 2011). In line with this, we observed significantly lower ABA level in endophyte-associated soybean seedlings as compared to endophyte-free plants. Our findings confirmed previously recorded lower amount of ABA in salt stressed endophytes associated plants (Khan et al., 2011). Still other studies suggested enhanced ABA production in plants inoculated with endophytic fungi (Hao et al., 2010). Hence, different groups of endophytic fungi may cause different effects on the endogenous ABA levels of plants as revealed by some earlier reports. Lower ABA in fungal associated plants suggests the involvement of GAs, as exogenous application of GA_3_ improved soybean salinity stress tolerance accompanied by low level of ABA (Hamayun et al., 2010). In salt affected plants, higher ABA is correlated with inhibition of leaf expansion and shoots development (Cramer and Quarrie, 2002). Next to ABA, GAs biosynthesis was significantly lower in salt affect seedlings. Recently, antagonism in the signaling of ABA and GA has been suggested (Cutler et al., 2010). GA-deficient plants are more susceptible to stress than those with higher levels of this hormone (Achard et al., 2006). Also, upregulation of gibberellin-deactivating gene, GA2ox7 in salt stressed *Arabidopsis* has been reported which represses growth for stress adaptation (Magome et al., 2008). The higher amount of GA_12_ in endophyte-treated plants under salinity stress elucidates the activation of GAs biosynthesis pathway, while higher production of GA_1_ and GA_4_ confirms plant growth maintenance during stress condition. Thus, by maintaining GAs and, therefore, growth under stress conditions, the endophyte is having a beneficial effect on the plant long-term survival. There are many previous reports showing the ameliorative effects of exogenous application of GAs (GA_1_/GA_4_) and IAA on plant growth under abiotic stress (Siddiqui et al., 2008; Arteca, 2013; Kaya et al., 2013), while information on the regulation of plant endogenous hormones in association with phytohormones producing endophytic fungi under abiotic stress conditions are scarce. Some physiological evidences demonstrate that plants infected with endophytic fungi often have a distinct advantage against biotic and abiotic stress over their endophyte-free counterparts (Rodriguez and Redman, 2008). Foliar application of GAs has been known for its role in plant stem elongation and mitigation of abiotic stress (Yang et al., 1996; Kaur et al., 1998), while the same was observed in current study that endophytes producing GAs rescued plants from the adverse effects of salinity stress.

**FIGURE 10:**
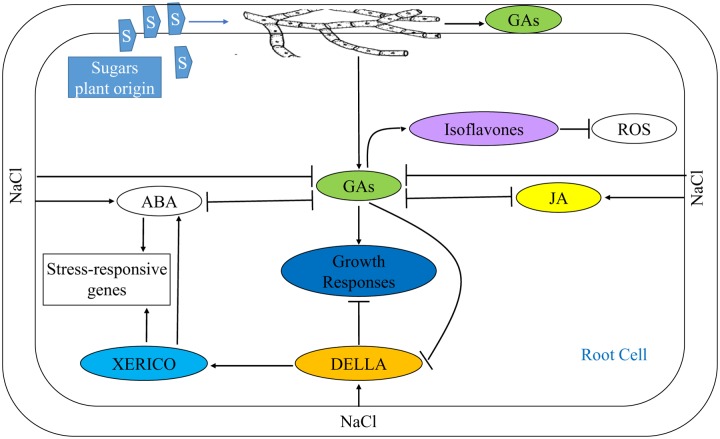
**Mitigation of NaCl stress in soybean seedlings by modulating phytohomones through application of an endophytic fungus *P. spadiceum* S-1-3**. NaCl stress suppresses GAs biosynthesis by enhanced biosynthesis of its antagonists, ABA and JA (or other independent mode), leading to retarded growth. Additionally, under NaCl stress, the stress responsive genes are induced either by enhanced biosynthesis of ABA or by DELLA protein in a XERICO dependent pathway. But in S-1-3 treated seedlings, the NaCl stress is alleviated by the boosted production of GAs that antagonize the biosynthesis of JA and ABA, and promotes biosynthesis isoflavones resulting in amelioration of different physiological and growth parameters of the stressed seedlings.

Restoration of ABA and GAs to their normal levels in salt affected plants associated with fungal endophytes demonstrates the mitigation of adverse effects of salt stress, probably by promoting plant growth and subsequently hindering build-up of DELLA proteins (**Figure 10**). ABA pathway is involved in plant responses to diverse abiotic and biotic inputs, and it is more likely that DELLA restraint provides a general mechanism for integration of plant growth responses to the environment. Salt stress also enhanced JA which shows the possible involvement of JA in the stress signal perception (Pedranzani et al., 2003). Inoculation of salt affected plants with fungal endophyte reduced endogenous JA to a level still higher than that of control plants advocating that alleviation of salt stress by fungal enodphyte is independent of JA signaling. JA signaling helps plants to combat against salt stress by enhancing the production of arginine decarboxylase, ribulose 1⋅5-bisphosphate carboxylase/oxygenase (Rubisco) activase and apoplastic invertase genes (Walia et al., 2007), that rationalizes the high level of JA in uninoculated salt stressed plants. High GAs in endophytes associated plants may act synergistically with SA in reducing JA signaling thereby making plant insensitive to JA. Additionally, enhanced GAs also promote SA signaling by degrading DELLA proteins (Navarro et al., 2008). Besides, GAs improve nodulation in soybean which may lead to stress alleviation (Hayashi et al., 2016).

Isoflavones are highly valued for human health, and soybean cultivars with higher isoflavones content have been developed over the past years (Phommalth et al., 2008). In addition, isoflavones are important regulators of root nodulation and plant defense in soybean and therefore, induction of isoflavones would be desirable for better field performance under stress (Algar et al., 2014). Our isolate AGH786 significantly enhanced isoflavone content of soybean under normal and salt stress conditions, thereby enhancing quality of soybean. Fungi are known to synthesized and release flavonoids which may be absorbed by plant, improving the endogenous pool of plant isoflavones (Qiu et al., 2010). Alternatively, plant enhanced isoflavones production might be due to increased activity of a key enzyme in isoflavone biosynthesis pathway (phenyl alanine ammonia lyase), under the influence of endophyte produced GAs (Barnes and Jones, 1984).

## Conclusion

Abiotic stress conditions prevailing in the environment are among the key constraints to agricultural production. This work reported a halophilic and halotolerant fungal strain, *P. spadiceum* AGH786 that not only boosted growth in soybean, but also helped it to resist salt stress. The fungal strain secreted bioactive GAs and free amino acids in the culture, modulated the endogenous phytohormones (GAs, ABA, JA, and SA) and enhanced isoflavones in inoculated seedlings intimating the ameliorative mechanism by which the fungal endophyte improved seedlings growth and their tolerance to salt stress. The reported strain is not only the ever first GAs producing endophytic basidiomycete, but is superior in terms of GA_3_ production to the so far reported most efficient GAs producing fungus *G. fujikori*. However, *G. fujikori* is still top GA_1_ producing fungus. This study opens an entry to unwrap this potential of basidiomycetes and urges for the use of such species as biofertilizers for enhancing productivity and food quality through real sustainable agriculture in saline soils.

## Author Contributions

MH: performed plant growth experiments and phytohormones determination. AH: isolated fungal endophytes and analyzed data statistically. SK: screened salt tolerance of the endophytes. AK: sequenced the isolates and identified the strains. MW: performed isoflavones determination. MI, AI, SJ, and GR: wrote draft manuscript. I-JL: supervised the study and finalized the draft manuscript.

## Conflict of Interest Statement

The authors declare that the research was conducted in the absence of any commercial or financial relationships that could be construed as a potential conflict of interest.

## Footnotes

1 http://www.ncbi.nlm.nih.gov/BLAST/
